# Molecular Characterization of Plasmids Harbored by Actinomycetes Isolated From the Great Salt Plains of Oklahoma Using PFGE and Next Generation Whole Genome Sequencing

**DOI:** 10.3389/fmicb.2018.02282

**Published:** 2018-09-28

**Authors:** Carolyn R. Cornell, Daya Marasini, Mohamed K. Fakhr

**Affiliations:** Department of Biological Science, The University of Tulsa, Tulsa, OK, United States

**Keywords:** actinomycetes, actinobacteria, linear plasmids, PFGE, Great Salt Plains, linear chromosome, next generation sequencing, extreme environments

## Abstract

One of the unique features of actinomycetes, especially the genus *Streptomyces*, is the presence of linear plasmids. These range in size from 12 to 600 kb, and are often termed mega-plasmids. While many of the genes involved in secondary metabolite production reside in clusters on the chromosome, several studies have identified biosynthetic clusters on large linear plasmids that produce important secondary metabolites, including antibiotics. In this study, Pulse Field Gel Electrophoresis (PFGE) was used to screen 176 actinomycete isolates for the presence of plasmids; these bacterial strains were previously isolated from the Great Salt Plains of Oklahoma. Seventy-eight of the 176 actinomycete isolates (44%) contained plasmids. Several strains contained more than one plasmid, accounting for a total of 109 plasmids. Ten isolates showed extrachromosomal DNA larger than 200 kb, thus falling into the category of mega-plasmids. A subset of plasmids from 55 isolates was treated with S1 nuclease to determine topology; all plasmids examined appeared to be linear and ranged from ~55 to 400 kb. Eleven isolates were chosen for Whole Genome Next Generation Sequencing. From the 11 sequenced isolates, seven plasmids were partially assembled. While the majority of the genes identified on the plasmids coded for hypothetical proteins, others coded for general functions, stress response, and antibiotic and heavy metal resistance. Draft genome sequences of two mega-plasmid-bearing *Streptomyces* sp. strains, BF-3 and 4F, revealed the presence of genes involved in antibiotic production, antibiotic, and heavy metal resistance, osmoregulation, and stress response, which likely facilitate their survival in this extreme halophilic environment. To our knowledge, this is the first study to explore plasmids harbored by actinomycetes isolated from the Great Salt Plains of Oklahoma.

## Introduction

Actinomycetales, an order within the phylum Actinobacteria, represent a large and diverse group of gram-positive bacteria that are known for being soil inhabitants with a high G+C content (Nett et al., 2009). The G+C content ranges widely among free-living Actinobacteria, with streptomycetes encoding over 70% G+C, corynebacteria averaging 54%, and the pathogen *Tropheryma whipplei* containing less than 50% (Chater and Chandra, 2006). Based on sequencing, plasmids within Actinobacteria have an average G+C content of 64.5% (Shintani et al., 2015). Actinobacteria provide a rich source of natural antibiotics, secondary metabolites for industrial processes, and host-vector systems (Connell, 2001). Generally, actinomycetes are saprophytic bacteria that remain in a semi-dormant state for most of their life cycle. Growth of hyphae tends to occur only when there is a large supply of nutrients available leading to the rapid production of spores (Mayfield et al., 1972). Actinomycetes with a more complex life cycle and structure, such as *Nocardia, Actinoplanes, Micromonospora, Streptovericillium, Streptomyces*, and *Saccharopolyspora*, have linear chromosomes (Lin et al., 1993; Reeves et al., 1998; Redenbach et al., 2000). The complexity of the life cycle does not play a role in the presence of linear plasmids, which have been found in actinomycetes with either type of chromosome topology. While the majority of actinomycetes studied to date are found in terrestrial environments, these organisms have been identified in a wide range of extreme environments and marine locations. These bacteria occupy environments where the soil has a high salt concentration such as the Great Salt Plains of Oklahoma, Antarctica, and several marine locations (Hotta et al., 1980; Lam, 2006; Encheva-Malinova et al., 2014; Gad, 2014). Another variation seen in the growth pattern of actinomycetes is in the wide range of temperatures where they occur. This is evident by the variety of environments they inhabit, ranging from psychrophilic to mesophilic conditions, and their presence and role during the high temperatures associated with composting (Kertesz and Thai, 2018).

For many years, eukaryotic organisms were presumed to be the only organisms containing linear chromosomes and telomeres (Bendich and Drlica, 2000). However, Hinnebusch and Tilly (1993) demonstrated that the linear structure of chromosomes possessing telomere-like structures is far more prevalent in bacteria than originally assumed, and some bacterial taxa may have the ability to change structure between linear and circular chromosomal forms. Plasmids were originally thought to be restricted to a circular topology; however, the discovery of the linear plasmid pSLA2s in *Streptomyces rochei* (Hayakawa et al., 1979) was followed by the identification of linear plasmids in other actinomycetes. These linear plasmids range from 12-600 kb (Kinashi et al., 1987; Sakaguchi, 1990) and are often called mega-plasmids, while the linear chromosomes in actinomycetes range from 8 to 10 Mbp (Hopwood, 2006). Although many of the genes involved in secondary metabolite production reside in clusters on the chromosome (Hopwood, 2007), several studies have identified biosynthetic clusters on the large linear plasmids that produce important secondary metabolites (Novakova et al., 2013). Linear plasmids are generally associated with specific bacterial strains and their roles remain cryptic in the majority of prokaryotes.

There are several significant gene clusters known for producing antibiotics on large linear plasmids. These plasmids include pSLA2-L in *S. rochei* (produces lankacidin and lankamycin) (Kinashi et al., 1994; Suwa et al., 2000), SCP1 in *Streptomyces coelicolor* A3(2) (methylenomycin) (Kirby and Hopwood, 1977), and pPZG103 in *Streptomyces rimosus* (oxytetracycline) (Gravius et al., 1994; Pandza et al., 1998). *S. coelicolor* A3(2) contains the best-characterized linear plasmid in the genus. Although the majority of large linear plasmids have been isolated from *Streptomyces* spp., other actinomycetes, including *Nocardia, Rhodococcus*, and *Mycobacterium*, also contain large extrachromosomal material (Kalkus et al., 1993; Dabrock et al., 1994; Picardeau and Vincent, 1997), thus demonstrating the vast potential that actinomycetes may contribute to the understanding of linear genetic elements and secondary metabolite production.

With over 900 described species, *Streptomyces* is the largest and most widely-studied genus of actinomycetes due to its extensive range of secondary metabolites, which are important in medical, veterinary, industrial, and agricultural processes (Chen et al., 2002). *Streptomyces* spp. form the foundation for 60% of the antibiotics used today and play a key role in the exchange of antibiotic resistance genes among bacteria (Davies, 1994, 1997). *Streptomyces* is one of the most complex forms of actinomycetes, growing as branching hyphal filaments of mycelium and reproducing by aerial branches moving upwards in chains of spores similar to fungi (Chater, 2006). Another extensive group in the phylum Actinobacteria, the genus *Nocardiopsis* is known for its pathogenicity, environmental versatility, and production of many secondary metabolites including apoptolidin (Kim et al., 1997), lipopeptide biosurfactants (Gandhimathi et al., 2009), methylpendolmycin (Sun et al., 1991), thiopeptides (Engelhardt et al., 2010), griseusin D (Li et al., 2007), and napthospironone A (Ding et al., 2010). Based on a study of 17 *Nocardiopsis* species, it appears their wide dispersal and ability to adapt to diverse conditions is based on their dynamic genomes (Li et al., 2013).

The Great Salt Plains of Oklahoma consists of ~11,000 acres (44.5 km^2^); it originated from an inland sea that left salt deposits. During the dry season, a crust of white salt forms on the surface due as a Permian brine rises to the surface and evaporates. During heavy rainfall, the salt deposits are dissolved and form saline ponds. The Great Salt Plains is considered an extreme environment due to salinity, UV exposure, and high temperatures. In a previous study, two actinomycete genera, *Streptomyces* (recovered from both vegetated and barren regions) and *Nocardiopsis* (recovered from vegetated regions) comprised the majority of the 200 actinomycete strains isolated (Gad, 2014). Most isolates tolerated a relatively high salt concentration with a few special cases surviving at 15% salinity (Gad, 2014).

In the current study, Pulsed Field Gel Electrophoresis (PFGE) was used to screen *Streptomyces* and *Nocardiopsis* strains isolated from the Great Salt Plains of Oklahoma for the presence of plasmids. The topology of the plasmids (e.g., linear or circular) was determined by PFGE and S1 nuclease treatment. Select plasmids were sequenced and analyzed along with their host chromosomes to identify potential secondary metabolites and stress tolerance genes that contribute to the survival of actinomycetes in the harsh environment of the Great Salt Plains.

## Materials and methods

### Plasmid detection by PFGE

Actinomycete isolates (*n* = 176) were previously collected from the Great Salt Plains of Oklahoma (Gad, 2014) and screened for the presence of plasmids using PFGE. The isolates included 24 *Nocardiopsis* spp., 74 *Streptomyces* spp., and 78 unidentified actinomycete spp. *Salmonella* Braenderup and *Escherichia coli* strain NCTC 50192 were used as standardized markers for sizing plasmids. Biosafety procedures were adhered to during the use of *Salmonella* Branderup. The CDC PulseNet protocol was used for sample preparation of the standardized markers. A protocol for actinomycete samples was developed based on protocols for molecular subtyping of *E. coli* O157:H7 and *Staphylococcus aureus* by PFGE (Redenbach et al., 2000; Marineo et al., 2005).

Actinomycete isolates were grown on starch nitrate glycerol (SNG) agar before transferring to liquid yeast malt extract (YEME) medium for 72 h at 28°C with constant shaking. Liquid cultures were adjusted to 1.5–2.0 OD at *A*_610_ in TE buffer. Markers for *E. coli* and *Salmonella* were grown overnight on TSA at 37°C, and the TE cell suspensions were made by using cells directly from the plates adjusted to an OD 0.8–1.0 at *A*_610_.

At least two plugs were made for each actinomycete isolate using disposable plug molds; plugs were also made for the molecular marker strains, *E. coli* NCTC 50192 and *Salmonella* Branderup. For the actinomycete samples, 400 μL of the cell suspension and 400 μL of 1% Seakem Gold Agarose were gently mixed using a pipette and heated to 50°C. Proteinase K (20 μL) was added to samples containing *E. coli* and *Salmonella*; afterwards suspensions were immediately transferred to plug molds and allowed to solidify at room temperature. Plugs were then transferred to tubes containing TE buffer and stored at 4°C until the cell lysis step.

Actinomycete agarose gel plugs were treated with 1 mg/mL of lysozyme for 2 h at 37°C with constant shaking. The lysozyme solution was then removed, and the plugs were then treated with 0.1 mg/mL of proteinase K at 54-55°C for 20–24 h. For *E. coli* strain NCTC 50192 and *S*. Braenderup, a cell lysis buffer (50 mM Tris: 50 mM EDTA, pH 8.0 + 1% sarcosyl) was prepared according to the PulseNet protocol. After lysis, the wash step consisted of adding 10–15 mL sterile millipore water and incubating samples at 50–55°C for 30 min with constant shaking; the water was removed, and the process was repeated. Four additional washes were conducted using the same conditions in TE buffer. *E. coli* strain NCTC 50192 and *S*. Braenderup agarose plugs were washed as described above, except water washes were 15–20 min. Agarose plugs were stored in TE buffer at 4°C until PFGE.

*S*. Braenderup agarose plugs were dissected into small slices (3–4 slices per plug), and two slices were treated with 50 units of *Xba*I enzyme (Promega, Madison, WI, USA) for at least 4 h at 37°C. Similarly, *E. coli* agarose plugs were cut into smaller slices and digested with S1 nuclease at 37°C for 45–60 min (Barton et al., 1995). All plugs were dried to remove any remaining TE buffer or restriction enzymes, and then attached to the comb in a 1% TBE agarose gel with three size markers. The gel was run in 0.5X TBE buffer in a cooling module set at 14°C for 16 h using the PulseNet oxacillin-resistant *S. aureus* protocol. The gel was stained using ethidium bromide and imaged using the Bio-Rad Gel DOC™ XR UV gel documentation system (BioRad, Hercules, CA, USA).

### Plasmid topology determination by PFGE

To determine whether plasmids of different sizes were linear or circular, S1 nuclease was used to treat all plasmids and examined by PFGE to determine plasmid topology (Barton et al., 1995). The plugs, along with two *S*. Braenderup standards, a standardized molecular weight marker, and uncut control, were set on the gel comb for comparison of plasmids from 55 different isolates.

### Next generation whole genome DNA sequencing and sequence analysis

Next generation sequencing was performed according to the Nextera® XT DNA Sample Preparation Guide (Illumina, San Diego, California, USA). Isolates were incubated in YEME broth for 72 h at 28°C with constant shaking. DNA was isolated with the Qiagen DNeasy Blood and Tissue Kit (Qiagen, Redwood City, California, USA). Eleven isolates were sequenced based on their varying plasmid sizes.

The Qubit® dsDNA BR Assay Kit and Qubit® Fluorometer 2.0 were used to adjust DNA concentrations to 0.2 ng/μL. The manufacturer's directions were followed for the Nextera XT Library Prep and the Nextera XT Index Kits; briefly, the main steps included tagmentation of input DNA and the addition of adapter sequences for PCR. After PCR, the short library fragments were removed using AMPure XP beads and the size of the amplicon pool was adjusted to >500 bp. Lastly, equal volumes of the size-normalized libraries were pooled, diluted with hybridization buffer, and heat-denatured before sequencing.

The MiSeq® v2 Reagent Kit was used for sequencing as recommended by the manufacturer (Illumina). Raw sequence data was analyzed using the CLC Genomics Workbench (Qiagen, Redwood City, California, USA) and the Genome Finishing Module plugin at CLC genomics. Sequence data were also analyzed using BLAST to determine if specific contigs were chromosomal or plasmid DNA. After a rough assembly of contigs using CLC Genomic Workbench, the assembled sequences were submitted to RAST (Rapid Annotation using Subsystem Technology) (Aziz et al., 2008) for annotation.

### Genbank accession numbers

The whole-genome shotgun sequences for *Streptomyces* sp. BF-3 (isolate SY-27-5-0%) and 4F (isolate SGR-27-4-5%) were deposited in GenBank under the accession nos. NACZ00000000 and NACY00000000, respectively. The draft genomes of the two isolates were previously reported as genome announcements (Cornell et al., 2018).

## Results

### Screening actinomycetes for plasmids using PFGE

**Figure 1:**
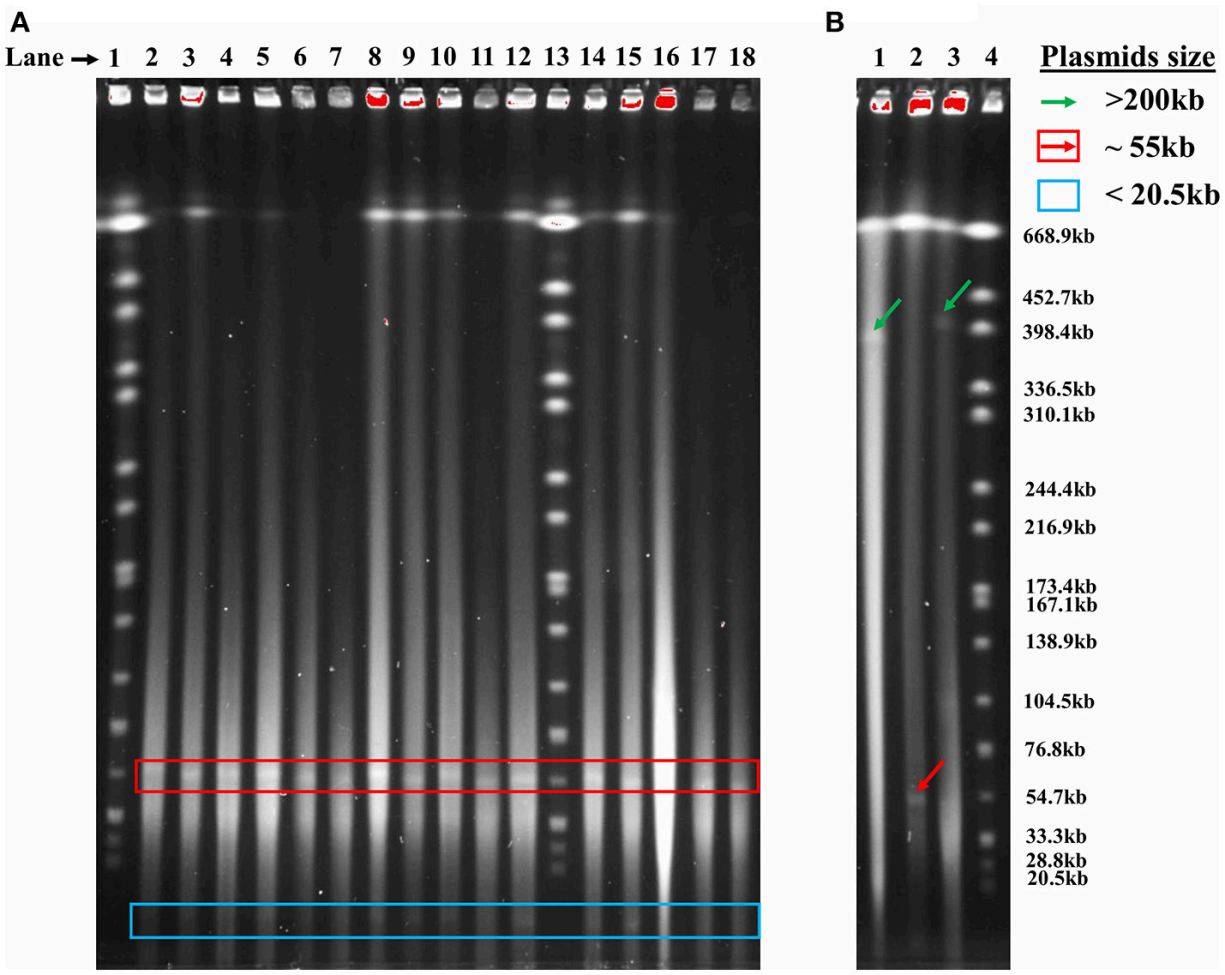
Detection of plasmids in actinomycete isolates using PFGE. **(A)** Lanes (left to right): (1) *S*. Branderup, (2) SY-20-2-5%, (3) SY-20-3-5%, (4) SY-20-5-10%, (5) SY-20-6-10%, (6) SY-20-7-5%, (7) SY-20-8-0%, (8) SY-20-9-0%, (9) SY-21-1-0%, (10) SY-21-2-0%, (11) SY-22-1-5%, (12) SY-22-2-5%, (13) *Salmonella* Branderup, (14) SY-24-1-0%, (15) SY-25-1-0%, (16) SY-25-5-0%, (17) SY-25-9-0%, (18) SY-25-4-10%. **(B)** Lanes (left to right): (1) SW-19-2-1-0%, (2) SW-20-2-10%, (3) SW-23-2-10%, (4) *S*. Branderup.

The 176 actinomycete isolates previously collected from the Great Salt Plains of Oklahoma (Gad, 2014) were screened by PFGE, and 78 (44%) contained plasmids. A total of 109 unique plasmids were identified with the majority of isolates containing one plasmid. Isolate SGR-27-6-0% has four plasmids, whereas five isolates (SY-21-3-5%, SY-26-6-5%, SGR-26-4-5%, SGR-27-1-0%, and SB-27-1-2-10%) each contained three plasmids. PFGE revealed the presence of two large plasmids (398 kb), several 55 kb plasmids, and a smaller plasmid estimated to be <20.5 kb (Figure 1). As can be seen in Figure 1, many samples contained plasmids of similar sizes and were often collected from the same or neighboring soil sample areas, which is reflected by the first number in the name of each isolate.

The plasmids detected by PFGE screening could be grouped into three main size ranges, >200, 71–200, and ≤ 70 kb (Table 1). Several isolates were previously identified using 16S rDNA sequencing (Gad, 2014) and assigned to *Streptomyces* or *Nocardiopsis*. No association could be determined between genus and plasmid size due to the limited number of genera screened. Ten isolates (Table 1) had mega-plasmids larger than 200 kb; most of these were similar in size. However, an exception was SGR-8-11-5%, which contained a unique 312 kb plasmid. The majority of the plasmids ranged from 71 to 200 kb (Table 1), and many were ~120 kb. Plasmids <70 kb were generally about 55 or <20 kb.

**Table 1:** Actinomycete isolates with plasmids with variable sizes (>200 kb shaded in gold, >70–200 Kb shaded in blue, and ≤ 70 kb shaded in green).

**Isolates used in the study**	**Closest Identification/Accession no**.	**Plasmids and their sizes**			
		**>200 kb**	**>70–200 kb**	**20–70 kb**	**<20 kb**
SGR-20-4-5%	Unidentified actinomycete isolate	405 kb	123 kb	–	–
SW-24-2-5%	Unidentified actinomycete isolate	400 kb	130 kb	–	–
SW-23-2-10%	gb|CP013142.1|*Streptomyces* sp. strain 4F	400 kb	–	–	–
SY-26-6-5%	gb|EU008821.1|*Streptomyces* sp. WBF11	398 kb	155 kb and 85 kb	–	–
SGR-27-6-0%	ref|NR_027223.1|*Streptomyces violaceolatus* strain DSM 40438	395 kb	148 kb and 136 kb	25 kb	–
SY-21-3-5%	gb|CP011522.1|*Streptomyces* sp. CFMR7	395 kb	140 kb	–	–
SGR-27-4-5%	gb*|*CP0131421.1*|Streptomyces* sp. strain 4F	395 kb	–	–	–
SY-27-3-5%	ref*|*NR_074635.1*|Nocardiopsis dassonvillei subsp. dassonvillei* DSM 43111 strain DSM 43111	380 kb	120 kb	–	–
SW-19-2-1-10%	gb*|*FJ982381.1*|Nocardiopsis sp*. FIRDI 009	380 kb	–	–	–
SGR-8-11-5%	gb*|*EU741237.1*|Streptomyces mutabilis* strain 13676F	312 kb	120 kb	–	–
SY-27-5-0%	gb|JN408756.1|*Streptomyces* sp. BF-3	–	193 kb	–	–
SGR-26-4-5%	gb|CP011522.1|*Streptomyces* sp. CFMR7	–	160 kb and 118 kb	65 kb	–
SY-27-2-10%	Unidentified actinomycete isolate	–	148 kb and 110 kb	–	–
SGR-26-1-0%	gb|GQ392058.1|*Streptomyces rochei* strain A-1	–	145 kb	–	–
SR-26-1-0%	emb|AM889494.1*|Streptomyces* sp. SHXFF-2	–	138 kb	–	–
SGR-14-4-0%	Unidentified actinomycete isolate	–	134 kb	–	–
SY-25-8-0%	gb|JX971566.1|*Streptomyces* sp. Cmuel-A718b	–	123 kb	–	–
SGR-27-4-0%	Unidentified actinomycete isolate	–	120 kb	52 kb	–
SGR-8-9-0%	gb*|*JN565291.1*|Streptomyces* sp. NPA1	–	120 kb	–	–
SGR-26-8-0%	ref|NR_025871.1|*Streptomyces tendae* strain ATCC 19812	–	120 kb	–	–
SGR-12-1-0%	Unidentified actinomycete isolate	–	120 kb	–	–
SGR-12-1'-0%	Unidentified actinomycete isolate	–	120 kb	–	–
SGR-20-1-0%	Unidentified actinomycete isolate	–	118 kb	–	–
SGR-19-4-0%	Unidentified actinomycete isolate	–	118 kb	–	–
SGR-20-5-0%	gb*|*JN565291.1*|Streptomyces* sp. NPA1	–	118 kb	–	–
SGR-24-7'-5%	Unidentified actinomycete isolate	–	116 kb	–	–
SGR-28-1'-4-0%	Unidentified actinomycete isolate	–	114 kb	–	–
SGN-19-7-10%	gb|EU741146.1|*Nocardiopsis* sp. 13647P	–	112 kb	–	–
SGR-26-2-0%	Unidentified actinomycete isolate	–	110 kb	–	<20 kb
SY-24-8-0%	gb|EU137870.1|*Streptomyces* sp. ALG5	–	108 kb	–	–
SW-25-1-5%	dbj*|*AB736324.1*|Nocardiopsis* sp. Sl115	–	104 kb	–	–
SY-25-3-5%	gb|JX971566.1|*Streptomyces* sp. Cmuel-A718b	–	104 kb	–	–
SB-27-1-2-10%	gb|GU130105.1|*Streptomyces* sp. 0614149 clone 105T3	–	104 kb	31 kb	<20 kb
SW-20-1-5%	gb*|*EU410477.2*|Nocardiopsis* sp. HM7	–	102 kb	–	–
SGR-19-8-5%	Unidentified actinomycete isolate	–	102 kb	–	–
SR-14-1-0%	ref|NR_025292.1|*Streptomyces somaliensis* strain DSM 40738	–	95 kb	–	–
SR-20-1-0%	gb|DQ849079.1|*Streptomyces* sp. CPC3	–	95 kb	–	–
SGN-19-2-0%	emb|FR845719.1|*Streptomyces venezuelae* ATCC 10712	–	95 kb	–	–
SB-3-2-5%	Unidentified actinomycete isolate	–	95 kb	–	–
SB-11-14-5%	Unidentified actinomycete isolate	–	95 kb	–	–
SB-27-1-3-10%	gb|GU130105.1|*Streptomyces* sp. 0614149 clone 105T3	–	95 kb	–	–
SBa-24-1'-0%	Unidentified actinomycete isolate	–	95 kb	–	–
SGR-8-8-0%	gb*|*JN936839.1*|Streptomyces* sp. CPE1	–	93 kb	–	–
SGR-22-4-3-5%	gb|AF429400.1|*Streptomyces* sp. VTT E-99-1336 (B329)	–	91 kb	–	–
SGR-27-1-0%	ref|NR_042309.1|*Streptomyces violaceorubidus* strain LMG 20319	–	90 kb	60 kb	–
SY-20-1-5%	Unidentified actinomycete isolate	–	90 kb	–	–
SGR-26-6-0%	Unidentified actinomycete isolate	–	85 kb	–	–
SGR-27-7-0%	Unidentified actinomycete isolate	–	81 kb	–	–
SY-21-5-5%	gb*|*AF540000.2*|Nocardiopsis tangguensis*	–	80 kb	–	–
SGR-8-10-0%	gb*|*DQ092377.1*|Streptomyces* sp. A-37	–	78 kb	–	–
SY-24-5-5%	gb*|*AF540000.2*|Nocardiopsis tangguensis*	–	77 kb	–	–
SGR-30-1-0%	Unidentified actinomycete isolate	–	76 kb	–	–
SGR-25-7-0%	gb|JN627185.1|*Streptomyces variabilis* strain A4-3	–	–	70 kb and 35 kb	–
SGR-24-3-0%	gb|JN627185.1|*Streptomyces variabilis* strain A4-3	–	–	64 kb	–
SW-21-2-10%	gb|FJ267618.1| *Streptomyces* sp. 216802	–	–	52 kb	–
SW-23-1-10%		–	–	52 kb	–
SY-26-3-10%		–	–	48 kb	–
SY-27-4-5%	ref*|*NR_074635.1*|Nocardiopsis dassonvillei subsp. dassonvillei* DSM 43111 strain DSM 43111	–	–	44 kb	–
SY-27-5-5%		–	–	~55 kb	–
SY-20-2-5%	gb|GQ213972.1|*Streptomyces* sp. NEAU	–	–	~55 kb	–
SY-20-3-5%	gb*|*AY299633.1*|Nocardiopsis sp. YIM 80251*	–	–	~55 kb	–
SY-20-5-10%	ref*|*NR_025589.1*| Nocardiopsis aegyptia* strain SNG49	–	–	~55 kb	–
SY-20-6-10%		–	–	~55 kb	–
SY-20-7-5%	gb|EF114310.2|*Streptomyces* sp. B5W22-2	–	–	~55 kb	–
SY-20-8-0%	gb|JF727260.1|*Streptomyces* sp. LYG-1	–	–	~55 kb	–
SY-20-9-0%		–	–	~55 kb	<20 kb
SY-21-1-0%	gb|JF727260.1|*Streptomyces* sp. LYG-1	–	–	~55 kb	<20 kb
SY-21-2-0%	gb|FJ267618.1|*Streptomyces* sp. 216802	–	–	~55 kb	<20 kb
SY-22-1-5%	gb|HQ392468.1|*Streptomyces* sp. OE53	–	–	~55 kb	<20 kb
SY-22-3-5%		–	–	~55 kb	<20 kb
SY-24-1-0%		–	–	~55 kb	<20 kb
SY-25-1-0%		–	–	~55 kb	<20 kb
SY-25-9-10%	gb*|*AY297777.1*|Nocardiopsis* sp. 10030	–	–	~55 kb	–
SY-26-4-10%		–	–	~55 kb	–
SW-20-2-10%	gb*|*AF540000.2*|Nocardiopsis tangguensis*	–	–	~55 kb	<20 kb
SY-24-6-0%	gb|FJ486453.1|*Streptomyces tendae* strain HBUM174966	–	–	~55 kb	–
SGR-8-6-0%		–	–	–	<20 kb

### Determining the topology of detected plasmids

**Figure 2:**
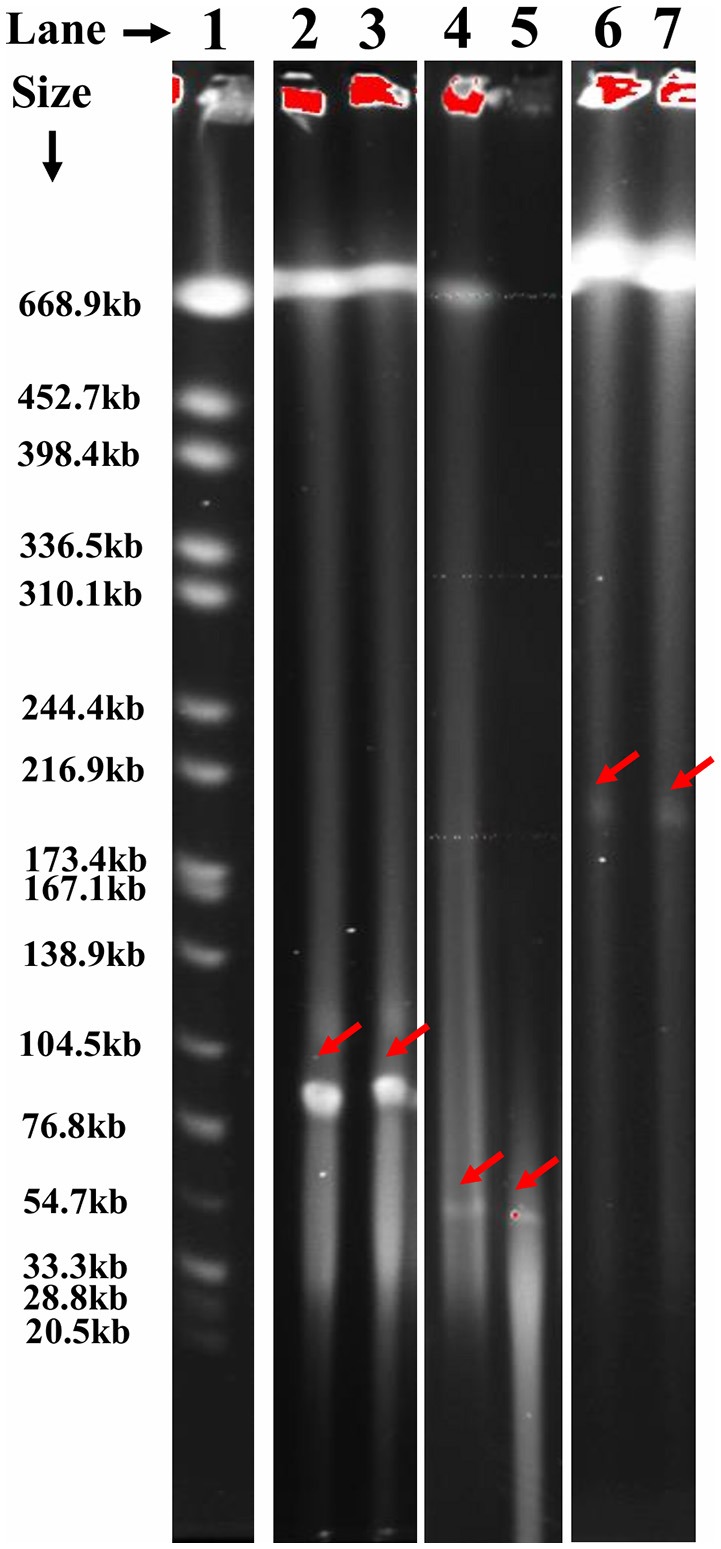
Determination of plasmids topology in actinomycete isolates untreated and treated with S1 nuclease using PFGE. Lanes (left to right): (1) *S*. Branderup, (2) untreated SY-20-1-5%, (3) treated SY-20-1-5%, (4) untreated SW-20-2-10%, (5) treated SW-20-2-10%, (6) untreated SY-27-5-0%, (7) treated SY-27-5-0%. No difference was noticed in the migration of the tested plasmids before and after treatment with S1 nuclease which indicates that these plasmids are linear. Plasmids are indicated by red arrows.

Large plasmids (>50 Kb) in actinomycetes are often linear, while smaller plasmids tend to be circular (Kinashi et al., 1987; Sakaguchi, 1990). To determine if the plasmids harbored by actinomycetes utilized in this study corresponded with previous findings, the topology of plasmids with a wide range of sizes (50–400 Kb) was examined. This was accomplished by S1 nuclease treatment of isolates followed by PFGE. Based on the screening of a subset of 55 isolates, all plasmids examined appeared to be linear (Figure 2). Figure 2 shows representative isolates with various plasmid sizes when subjected to PFGE before and after treatment with S1 nuclease. No difference was noticed in the migration of the tested plasmids before and after treatment with S1 nuclease which indicates that these plasmids are linear.

### Sequence analysis of plasmid-bearing isolates and annotation of selected genes

Next generation sequencing was performed on a subset of eleven isolates (three *Nocardiopsis* spp. and eight *Streptomyces* spp.) containing plasmids of various sizes (Table 2). Assembled contigs were analyzed using BLASTN to determine whether sequences were plasmid-borne or chromosomal. Due to the limited data on actinomycete plasmids in GenBank, only partial plasmid sequences could be constructed for several isolates.

**Table 2:** Actinomycete isolates and plasmids used for next generation sequencing.

**Isolate**	**Species**	**Plasmid 1 (kb)**	**Plasmid 2 (kb)**	**Plasmid 3 (kb)**
SW-23-2-10%	*Streptomyces* sp.	400		
SY-21-3-5%	*Streptomyces* sp.	395	140	130
SGR-27-4-5%	*Streptomyces* sp.	395		
SW-19-2-1-10%	*Nocardiopsis* sp.	380		
SY-27-5-0%	*Streptomyces* sp.	193		
SGR-26-4-5%	*Streptomyces* sp.	160	118	65
SB-27-1-2-10%	*Streptomyces* sp.	104		
SGR-27-1-0%	*Streptomyces violaceorubidus*	90	75	60
SY-21-5-5%	*Nocardiopsis tangguensis*	80		
SY-21-2-0%	*Streptomyces* sp.	~55	<20	
SW-20-2-10%	*Nocardiopsis tangguensis*	~55	<20	

The partially constructed plasmid sequences were analyzed using RAST to identify genes of interest (Table 3). The majority of the plasmid sequences were classified as hypothetical proteins along with proteins used for general cellular functions. Some of the plasmid sequences showed the presence of genes involved in plasmid transfer, multidrug transport system, and synthesis of antibiotics (Table 3). Since very few genes could be defined, the draft Whole Genome Sequences of two isolates were used to construct their chromosomes to identify genes of interest. Genes of interest that were identified on these two chromosomes include those needed for survival in extreme environments, and genes involved in antibiotic biosynthesis/resistance and heavy metal resistance. The chromosome of isolate SGR-27-4-5%, *Streptomyces* sp. strain 4F, was constructed using the complete genome of *Streptomyces lividans* TK4 (GenBank accession no. CP009124). The chromosome of isolate SY-27-5-0%, *Streptomyces sp*. BF-3, was constructed using the complete *Streptomyces globisporus* C-1027 genome (accession no. CP013738). Table 4 includes a list of notable genes encoded on the chromosome of *Streptomyces* sp. strain 4F, (isolate SGR-27-4-5%). The genes of interest on the chromosome of *Streptomyces* sp. BF-3, (isolate SY-27-5-0%) are listed in Table 5. Both isolates contained genes coding for universal stress proteins, resistance to cobalt-cadmium-zinc, arsenic, and tellurium, vitamin and antibiotic production, and antibiotic resistance. The draft genome sequences of *Streptomyces* sp. strains BF-3 (SY-27-5-0%) and 4F (SGR-27-4-5%) were previously reported as genome announcements and were 7,950,134 and 7,550,992 bp, respectively (Cornell et al., 2018).

**Table 3:** Genes present on plasmids harbored by actinomycetes sequenced in this study.

**Isolate**	**Genes(s) Present**	Gene Size (bp)^*^	**Accession Number**
SW-19-2-10%	Transcriptional regulator (2)^**^	681	WP_018521698.1
	Chromosome (plasmid) partitioning protein ParA and ParB	1248	WP_010064700.1
SW-20-2-10%	Transfer protein TraSA	1911	SBV06812.1
	Transcriptional regulator	249	WP_024127206.1
	Aclacinomycin oxidoreductase	1638	OCC08800.1
	Universal stress protein family	393	WP_032765329.1
	WhiB-like transcription regulator	906	WP_056704985.1
	Plasmid partitioning protein ParA	660	WP_012821767.1
SY-21-2-10%	Chromosome (plasmid) partitioning protein ParA and ParB	996	ODA70275.1
	RNA polymerase σ^70^, ECF subfamily	660	WP_043441614.1
SY-21-5-5%	Transcriptional regulator, MerR family	399	WP_032765254.1
	RNA polymerase σ^70^, ECF subfamily	591	EFE74770.1
	Chromosome (plasmid) partitioning protein ParA	996	ODA70275.1
SY-27-5-0%	ABC-type multidrug transport system ATPase and permease components (2)^**^	1746	SCF58705.1
	Transcriptional regulatory protein (7)^**^	489	SCF65435.1
SGR-27-1-0%	Putative integral membrane plasmid transfer protein	450	KOV81945.1
	Transcriptional regulator (3)^**^	741	WP_024888670.1
SGR-27-4-5%	Chromosome (plasmid) partitioning protein ParA and ParB	1155	WP_020945073.1
	Putative plasmid transfer protein, putative transfer protein SpdA, mobile element transfer protein SpdB	1356	WP_030399944.1
	Transcriptional regulator (6)^**^	369	WP_031169591.1
	Tellurium resistance protein TerD (2)^**^	576	WP_020114594.1
	Cold shock protein	204	WP_019324834.1
	Universal stress protein family (3)^**^	939	WP_030975022.1
	ABC-type multidrug transport system, permease component; putative drug exporters of the RND superfamily	783	SBU94049.1
SB-27-2-10%	Transcriptional regulator (3)^**^	831	WP_011039338.1
	Polymyxin synthetase PmxB	288	WP_005244517.1

* *Gene size shown is representing the coding region*.

** *Number of genes present are for genes of the same function. The size of the gene shown is for one representative gene with the corresponding accession number shown*.

**Table 4:** Chromosomally-encoded genes in *Streptomyces* sp. strain 4F (isolate SGR-27-4-5%).

**Gene(s) of Interest**	# of Genes Present^**^	Gene Size (bp)^*^	**Accession Number**
Mobile element protein (mobile element transfer protein SpdB)	11	402	WP_054100445
Universal stress protein family	4	906	WP_019327273
Osmoregulation	3	477	SBT92325
Ectoine biosynthesis and regulation	5	900	WP_052841845.1
Choline and betaine uptake and betaine biosynthesis	10	666	WP_020271580
Heat shock protein (heat shock DnaK gene cluster, extended: hypothetical radical SAM family enzyme, coproporphyrinogen III oxidase, oxygen-independent, translation termination factors, bacterial: tmRNA-binding protein SmpB, heat shock protein 60 family chaperone GroEL, heat shock protein 60 family co-chaperone GroEL, chaperone protein DnaK, chaperone protein DnaJ, heat shock protein GrpE, heat-inducible transcription repressor HrcA)	14	438	WP_043376487
Alkaline shock protein 23	3	498	WP_037909982
Cold shock protein (cold shock protein CspD, cold shock protein CspA, cold shock protein CspG)	4	204	WP_023586105
Cobalt-zinc-cadmium resistance: transcriptional regulator, MerR family (cobalt-zinc-cadmium resistance protein CzcD, DNA-binding heavy metal response regulator, probable Co/Zn/Cd efflux system membrane fusion protein)	7	1185	WP_043381403
Tellurium resistance protein (TerD, TerA)	6	456	WP_043371552
Arsenic resistance: arsenical pump-driving ATPase (arsenical-resistance protein ACR3, arsenical resistance operon repressor, arsenate reductase)	7	978	WP_043371752
Uptake of selenate and selenite: (various polyols ABC transporter, permease component 2, ATP-binding component 1)	3	783	WP_011030595
Lantibiotic ABC transporter	1	807	CCQ18686
Putative drug exporters of the RND superfamily	3	1398	WP_016327935
Salicylate and gentisate catabolism, salicylate ester degradation: salicylate hydroxylase (EC 1.14.13.1)	1	1716	WP_016327418
ABC-type multidrug transport system, permease component	1	783	WP_032757890
Multidrug resistance protein B	1	2097	WP_055419087
Tetracycline resistance, ribosome protection type, translation elongation factor G family (tetracycline resistance protein)	2	2128	WP_043381067
Vancomycin response regulator VanR	2	2808	WP_043374748
Protein involved in biosynthesis of mitomycin antibiotics/polyketide fumonisin	1	1164	WP_043377507
Putative penicillin acylase (penicillin amidase family protein)	2	2808	WP_043374748
Pyridoxin (vitamin B6) biosynthesis, thiamine biosynthesis: 1-deoxy-D-xylulose 5-phosphate synthase (pyridoxamine 5'-phosphate oxidase, pyridoxine biosynthesis glutamine amidotransferase, synthase subunit (EC 2.4.2.-), CBSS-1806.1.peg.1285, pyridoxine biosynthesis glutamine amidotransferase, glutaminase subunit (EC 2.4.2.-)	10	1929	WP_046249165
Folate biosynthesis: thymidylate synthase ThyX (EC 2.1.1.-) (dihydrofolate synthase, EC 6.3.2.12), folylpolyglutamate synthase (EC 6.3.2.17), folate biosynthesis cluster: FIG027937: secreted protein)	3	741	WP_043379203
Menaquinone via futalosine step 3 (gene SCO4494, often clustered with other genes in menaquinone via futalosine pathway, AsnC-family transcriptional regulator SCO4493 in menaquinone synthesis cluster, menaquinone via futalosine polyprenyltransferase (MenA homolog), UbiD family decarboxylase associated with menaquinone via futalosine)	5	1200	WP_043381260
Antibiotic biosynthesis monooxygenase	1	348	WP_043383968
Xylose utilization: Endo-1,4-β-xylanase A precursor (EC 3.2.1.8) (xylose utilization: α-xylosidase, xylulose kinase, xylose-responsive transcription regulator, ROK family, possible alpha-xyloside ABC transporter, ATP-binding component, possible alpha-xyloside ABC transporter, permease component)	7	1389	WP_030969144.1
Chitin and N-acetylglucosamine utilization: chitinase	1	1812	WP_030971667

* *Gene size shown is representing the coding region*.

** *Number of genes present are for genes of the same function. The size of the gene shown is for one representative gene with the corresponding accession number shown*.

**Table 5:** Chromosomally-encoded genes in Streptomyces sp. BF-3 (isolate SY-27-5-0%).

**Gene(s) of Interest**	# of Genes Present^**^	Gene Size (bp)^*^	**Accession Number**
Mobile protein element	14	414	WP_032761331
Osmoregulation	3	846	SBU96315
Ectoine biosynthesis and regulation	5	894	WP_032761401
Choline and betaine uptake and betaine biosynthesis	10	2622	WP_010070215
Universal stress protein	9	864	WP_030329972
Alkaline shock protein 23	5	543	WP_032767011
Cold shock, CspA family of proteins: cold shock proteins CspA and CspC	5	203	WP_003967102
Heat shock protein 60 family co-chaperone GroES	2	258	WP_003966899
Arsenic resistance: arsenic efflux pump protein, pump-driving ATPase, operon repressor, protein ACR3	8	1158	AGK80249
Tellurium resistance protein (TerD and TerA)	9	456	WP_006125865
Cobalt-zinc-cadmium resistance: Transcriptional regulator, MerR family, probable Co/Zn/Cd efflux system membrane fusion protein, DNA-binding heavy metal response regulator, cobalt-zinc-cadmium resistance protein CzcD	8	345	WP_032775780
Copper resistance protein, multicopper oxidase, protein D, copper-translocating P-type ATPase	5	1541	ESU48807
Tetracycline resistance protein	1	1257	WP_032766138
Phosphotransferase (aminonucleoside antibiotic resistance)	1	564	WP_032779818
Putative bicyclomycin resistance protein	1	1335	WP_032761820
Cobalamin synthesis: cobalamin synthase, threonine kinase (B12 biosynthesis), nicotinate-nucleotide-dimethylbenzimidazole phosphoribosyltransferase, cobalt-precorrin-4 C11-methyltransferase	6	783	EGE44956
Folate biosynthesis: dihydrofolate synthase (EC 6.3.2.12), folylpolyglutamate synthase (EC 6.3.2.17), thymidylate synthase ThyX	9	1521	WP_032779143
Pyridoxin (vitamin B6) biosynthesis: predicted transcriptional regulator of pyridoxine metabolism, pyridoxamine 5'-phosphate oxidase (EC 1.4.3.5), pyridoxine biosynthesis glutamine amidotransferase, synthase subunit and glutaminase subunit	7	1182	WP_032778505
Putative toxic cation resistance protein	1	768	WP_050505692
RND multidrug efflux transporter; acriflavin resistance protein	1	3156	WP_010057790
Phosphotransferase (aminonucleoside antibiotic resistance)	1	564	WP_032779818
Drug resistance transporter, EmrB/QacA family	1	1676	WP_010059785
Penicillin amidase precursor (EC 3.5.1.11), putative penicillin-binding protein, β-lactamase class C and other penicillin binding proteins	3	2745	WP_032781722
Streptothricin resistance: streptothricin acetyltransferase, *Streptomyces lavendulae* type	1	567	WP_032776846

* *Gene size shown is representing the coding region*.

** *Number of genes present are for genes of the same function. The size of the gene shown is for one representative gene with the corresponding accession number shown*.

## Discussion

Actinomycetes are ubiquitous in soils and generally occur as spores in soils until nutrients become available (Mayfield et al., 1972; Williams et al., 1984). Although prokaryotes were originally considered to contain only circular replicons, reports of linear DNA molecules are becoming more common (Hinnebusch and Tilly, 1993; Chen, 1996), particularly in actinomycetes where mega-plasmids are involved in antibiotic production (Kinashi and Shimaji, 1987; Kinashi et al., 1987). In this study, ten actinomycete isolates contained plasmids exceeding 200 kb. Previous studies indicate that plasmids in this size class are generally linear. To confirm this, 55 different isolates possessing one or more plasmids were treated with S1 nuclease and compared with an untreated isolate. The analysis showed that all plasmids ≥55 kb were linear. Results from next generation sequencing indicated that the plasmids and chromosomes in this study could be partially assembled with linear plasmid and chromosomal references. Thus, the results of PFGE analysis and next generation sequencing indicate a linear topology for all large plasmids (>50 Kb) and chromosomes in this study.

Microorganisms must adapt to their physiological environment to function optimally, and the ability to overcome stressful conditions is imperative. Actinomycete isolates from the Great Salt Plains possess a range of genes encoding universal stress response proteins and heat, cold and alkaline shock proteins. In general, these stress response genes are used for survival in harsh environmental conditions. In Oklahoma, temperatures can range from subzero to 37°C and higher, which explains the importance of the temperature shock genes identified on plasmids and chromosomes in this study (Tables 3–5). The study location also exhibits extreme fluctuations in salinity, and actinomycetes are likely to survive due to the abundance of universal stress proteins and osmoregulatory proteins. Strategies for maintaining osmoregulation range from accumulating inorganic salts to controlling the concentration of compatible solutes. The chromosomes of the two actinomycetes characterized in this study contain genes encoding choline and glycine betaine aldehyde; these are precursors to glycine betaine, which protects plants from osmotic stress (Tables 4, 5). The two actinomycete chromosomes also contain genes encoding ectoine biosynthetic proteins (Tables 4, 5). Ectoine is an osmolyte found in many halophilic and halotolerant microorganisms including Actinobacteria (Malin and Ladpidot, 1996). Therefore, actinomycetes utilize a variety of methods to cope with the changing salt concentrations at the Great Salt Plains, such as the biosynthesis of solutes, uptake of osmoprotectants, and regulation of water transport.

Although metals play important roles in bacterial metabolism, the majority are nonessential and/or toxic. It has been hypothesized that the evolution of heavy metal resistance is ancient and was a response of prokaryotes to metal pollution. Resistance to metals is mediated through a variety of mechanisms that are borne on plasmids, transposons, and chromosomes. Bacteria have at least six metal resistance strategies, including intra- and extra-cellular sequestration, enzymatic detoxification, active transport efflux pumps, reduced sensitivity of cellular targets to metal ions, and exclusion by permeability barriers (Bruins et al., 2000). Metal resistance via plasmid-encoded genes evolved as a method to deal with toxic elements (Nies, 1992); related systems have been identified in the chromosome of *Bacillus* spp. and *E. coli* (Silver, 1996). Cadmium and zinc are chemically related, and cobalt is known to have affinity for zinc binding sites, supporting the idea that one efflux system is used to transport the cations of all three metals (Schneider-Bernlohr et al., 1988; Nies and Silver, 1989). In the current study, genes for cobalt-zinc-cadmium resistance were present and chromosomally-encoded in both actinomycete strains (Tables 4, 5).

The chromosomes of the two actinomycetes studied herein also encode arsenic and tellurium resistance. Tellurium resistance genes were also identified on plasmids sequenced in this study (Table 3). Tellurium is toxic to most bacteria and its resistance is frequently associated with resistance to arsenic, mercury, or silver compounds (Summers and Jacoby, 1977). Arsenic is one of the most abundant toxic metals in the environment and originates from both geochemical and anthropogenic sources (Mukhopadhyay et al., 2002). Arsenate is structural analog of phosphate; it can enter the cell using the phosphate transport system and interfere with phosphorylation reactions. In the present study, the genes encoding tellurium and arsenic resistance were chromosomally-encoded in the two *Streptomyces* spp. (Tables 4, 5). Genes for the uptake of selenate and selenite were also present on the linear chromosome of *Streptomyces* sp. strain 4F. The Great Salt Plains Lake is the only known area in the world where selenite crystals have been documented. This occurs when concentrated saline water combines with gypsum, thus promoting crystal growth with an hourglass shaped sand inclusion. Tellurite and selenite are chemically related to sulfate, and Scala and Williams (1963) proposed that these compounds could be potentially reduced by the sulfate pathway. Furthermore, tellurite may have the ability to replace sulfur in a number of cellular functions (Summers and Jacoby, 1977), leading to detrimental effects on bacterial cells.

Copper resistance genes were detected in *Streptomyces* sp. BF3 but not in *Streptomyces* sp. strain 4F (Table 4, 5). The *cop* resistance operon has been well-characterized in the chromosome of the gram-positive bacterium *Enterococcus hirae* and in the plasmids of *Pseudomonas* (Cooksey, 1994), *Xanthomonas* (Lee et al., 1994), and *E. coli* (Brown et al., 1994, 1995). Based on a contamination study at the Salt Plains National Wildlife Refuge from 1990-2001, there were detectable levels of selenium and copper in water, sediment, and fish (Martin, 2002). Cadmium and zinc were in water samples, while other researchers found arsenic, cadmium, and zinc in sediments (Logan and Morgan, 1990; Persaud et al., 1993). The occurrence of these elements in the water and sediments of the Great Salt Plains explains the presence of the varied heavy metal resistance systems encoded by the two *Streptomyces* spp.

Many of the antibiotics produced commercially today are produced by *Streptomyces* and related bacteria, which encode genes for both antibiotic biosynthesis and resistance. With respect to antibiotic resistance, both *Streptomyces* spp. in this study had chromosomal genes conferring resistance to tetracycline, bicyclomycin, streptothricin, as well as drug exporters and multidrug transport systems. Multidrug transport system and drug exporters were also found on plasmids sequenced in this study (Table 3). While tetracycline and bicyclomycin are broad-spectrum antibiotics, streptothricin is not as commonly used but has the ability to inhibit prokaryotic protein biosynthesis. The vancomycin response regulator VanR, which was detected in the sequenced isolates, is part of the two-component signal transduction system, VanRS (Hutchings et al., 2006). Multi-drug resistant bacteria are increasingly common and such resistance generally occurs due to the accumulation of transferable resistance genes encoded by transposons or plasmids or is mediated by multidrug efflux pumps that transport multiple compounds (Nikadio, 2009). It is known that a major source of resistance to particular drugs, especially tetracycline, is from multidrug efflux pumps. A study by D'Costa et al. (2006) examined antibiotic resistance in *Streptomyces* spp. and relatives isolated from soil samples and showed that 60–100% of the isolates were resistant to several antibiotics.

Actinomycetes are famous for antibiotic production and are the source of over half of the natural antibiotics that are used today. In the present study, genes coding for proteins involved in the biosynthesis of mitomycin antibiotics/polyketide fumonisin, putative penicillin acylase (penicillin amidase family protein), and antibiotic biosynthesis monooxygenase were evident in *Streptomyces* sp. strain 4F (Table 4). Polymyxin synthetase and aclacinomycin oxidoreductase genes were found on some of the plasmids sequenced in this study (Table 3). *Streptomyces* spp. BF-3 possesses genes for polymyxin synthetase PmxB, antibiotic biosynthesis monooxygenase, and a penicillin amidase precursor. Penicillin amidase functions in the biosynthesis of penicillin by catalyzing the hydrolysis of amide bonds in ampicillin, penicillin G, and penicillin V. Monooxygenases generally perform the hydroxylation of intermediates in the antibiotic biosynthesis pathways (O'Keefe and Harder, 1991). The two bacterial strains characterized in this study also have the potential to produce vitamins including folate, vitamin B-12 (cobalamin), vitamin B-6 (pyridoxine), vitamin K-2 (menaquinone), and vitamin K-1 (phylloquinone) (Tables 4, 5).

In conclusion, actinomycetes isolated from the Great Salt Plains of Oklahoma harbor a variety of plasmids, including mega-plasmids, which encode genes potentially involved in adaptation to this extreme environment. Further studies are underway to determine if these plasmids are similar and can be conjugally transferred to other actinomycetes. Draft genome sequences of two megaplasmid-bearing *Streptomyces* sp. strains, BF-3, and 4F, revealed the presence of genes involved in antibiotic production, antibiotic, and heavy metal resistance, osmoregulation, and the stress response, which facilitates their survival in this extreme halophilic environment. To our knowledge, this is the first study to explore plasmids harbored by actinomycetes isolated from the Great Salt Plains of Oklahoma.

## Author contributions

MF prepared the research idea and design of the manuscript. CC and DM performed the experimental procedures. CC and MF prepared the manuscript.

### Conflict of interest statement

The authors declare that the research was conducted in the absence of any commercial or financial relationships that could be construed as a potential conflict of interest.

## References

[B1] Aziz R. K. Bartels D. Best A. A. DeJongh M. Disz T. Edwards R. A.. (2008). The RAST server: rapid annotations using subsystems technology. BMC Genomics 9:75. 10.1186/1471-2164-9-75 18261238 PMC2265698

[B2] Barton B. M. Harding G. P. Zuccarelli A. J. (1995). A general method for detecting and sizing large linear plasmids. Anal. Biochem. 226, 235–240. 10.1006/abio.1995.1220 7793624

[B3] Bendich A. J. Drlica K. (2000). Prokaryotic and eukaryotic chromosomes: what's the difference? Bioessays 22, 481–486. 10.1002/(SICI)1521-1878(200005)22:5<481::AID-BIES10>3.0.CO;2-T 10797488

[B4] Brown N. L. Barrett S. R. Camakaris J. Lee B. T. Rouch D. A. (1995). Molecular genetics and transport analysis of the copper-resistance determinant (pco) from *Escherichia coli* plasmid pRJ1004. Mol. Microbiol. 17, 1153–66. 8594334 10.1111/j.1365-2958.1995.mmi_17061153.x

[B5] Brown N. L. Lee B. T. O. Silver S. (1994). Bacterial transport of resistance to copper, in Metal Ions in Biological Systems, Vol. 30, eds Seigel H. Seigel A. (New York, NY: Marcel Dekker), 405–434.

[B6] Bruins M. R. Kapil S. Oehme F. W. (2000). Microbial resistance to metals in the environment. Ecotoxicol. Environ. Saf. 45, 198–207. 10.1006/eesa.1999.1860 10702338

[B7] Chater K. F. (2006). *Streptomyces* inside-out: a new perspective on the bacteria that provide us with antibiotics. Phil. Trans. R. Soc. B 361, 761–768. 10.1098/rstb.2005.1758 16627293 PMC1609407

[B8] Chater K. F. Chandra G. (2006). The evolution of development in *Streptomyces* analyzed by genome comparisons. FEMS Microbiol. Rev. 30, 651–672. 10.1111/j.1574-6976.2006.00033.x 16911038

[B9] Chen C. W. (1996). Complications and implications of linear bacterial chromosome. Trends Genet. 12, 192–296. 10.1016/0168-9525(96)30014-0 8984735

[B10] Chen C. W. Huang C. H. Lee H. H. Tsai H. H. Kirby R. (2002). Once the circle has been broken: dynamics and evolution of *Streptomyces* chromosomes. Trends Genet. 18, 522–529. 10.1016/S0168-9525(02)02752-X 12350342

[B11] Connell N. D. (2001). Expression systems for use in actinomycetes and related organisms. Curr. Opin. Biotechnol. 28, 241–256. 10.1016/S0958-1669(00)00243-3 11604318

[B12] Cooksey D. A. (1994). Molecular mechanism of copper resistance and accumulation in bacteria. FEMS Microbiol. Rev. 14, 381–386. 10.1111/j.1574-6976.1994.tb00112.x 7917425

[B13] Cornell C. R. D. Marasini M. K. Fakhr (2018). Draft Genome sequences of mega-plasmid bearing *Streptomyces* sp. strains BF-3 and 4F isolated from the great salt plains of Oklahoma. Genome Announc. 6, e00208–18. 10.1128/genomeA.00208-18 29622611 PMC5887023

[B14] Dabrock B. Kessler M. Averhoff B. Gottschalk G. (1994). Identification and characterization of transmissible linear plasmid from *Rhodococcus* erythropolis BD2 that encodes isopropylbenze and trichloroethane catabolism. App. Environ. Microbiol. 60, 853–860. 10.1128/aem.60.3.853-860.1994 PMC201402 8161179

[B15] Davies J. (1994). New pathogens and old resistance genes. Microbiolgia 10, 9–12. 7946130

[B16] Davies J. E. (1997). Origins, acquisitions and dissemination of antibiotics resistance determinants. Ciba Found. Symp. 207, 15–35. 9189632

[B17] D'Costa V. M. McGrann K. M. Hughes D. W. Wright G. D. (2006). Sampling the antibiotic resistome. Science 311, 374–377. 10.1126/science.1120800 16424339

[B18] Ding Z. G. Li M. G. Zhao J. Y. Ren J. Huang R.. (2010). Naphthospironone A: an unprecedented and highly functionalized polycyclic metabolite from an alkaline mine waste extremophile. Chem. Eur. J. 16, 3902–3905. 10.1002/chem.200903198 20209526

[B19] Encheva-Malinova M. Stoyanova M. Avramova H. Pavlova Y. Gocheva B. Ivanova I.. (2014). Antibacterial potential of streptomycete strains from Antarctic soils. Biotechnol. Biotechnol. Equip. 28, 721–727. 10.1080/13102818.2014.947066 26019556 PMC4434119

[B20] Engelhardt K. Degnes K. F. Kemmler M. Bredholt H. Fjaervik E.. (2010). Production of a new thiopeptide antibiotic, TP-1161, by a marine Nocardiopsis species. Appl. Environ. Microbiol. 76, 4969–4976. 10.1128/AEM.00741-10 20562278 PMC2916467

[B21] Gad A. H. (2014). Bacterial Diversity at the Great Salt Plains of Oklahoma. Doctoral dissertation The University of Tulsa.

[B22] Gandhimathi R. Seghal Kiran G. Hema T. A. Selvin J. Rajeetha Raviji T.. (2009). Production and characterization of lipopeptide biosurfactant by a spongeassociated marine actinomycetes Nocardiopsis alba MSA10. Bioprocess Biosyst. Eng. 32, 825–835. 10.1007/s00449-009-0309-x 19288138

[B23] Gravius B. Glocker D. Pigac J. Pandza K. Hranueli D. Cullum J. (1994). The 387 kb linear plasmid pPZG101 of *Streptomyces rimosus* and its interactions with the chromosome. Microbiology 140, 2271–2277. 10.1099/13500872-140-9-2271 7952179

[B24] Hayakawa T. Tanaka T. Sakagushi K. Otake N. Yonehara H. (1979). A linear plasmid-like DNA in *Streptomyces* sp. producing lankacidin group antibiotics. J. Gen. Appl. Microbiol. 25, 255–260. 10.2323/jgam.25.255

[B25] Hinnebusch J. Tilly K. (1993). Linear plasmids and chromosomes in bacteria. Molec. Microbiol. 10, 917–922. 10.1111/j.1365-2958.1993.tb00963.x 7934868

[B26] Hopwood D. A. (2006). Soil to genomics, the *Streptomyces* chromosome. Ann. Rev. Genet. 40, 1–23. 10.1146/annurev.genet.40.110405.090639 16761950

[B27] Hopwood D. A. (2007). Streptomyces in Nature and Medicine: the Antibiotic Markers. New York, NY: Oxford University Press.

[B28] Hotta K. Satto N. Okami Y. (1980). Studies on new aminoglycoside antitiotics, istamycins, from an actinomycetes isolated from a marine environment. J. Antibiot. 33, 1502–1509. 10.7164/antibiotics.33.1502 7251490

[B29] Hutchings M. I. Hong H. J. Buttner M. J. (2006). The vancomycin resistance VanRS two-component signal transduction system of *Streptomyces coelicolor*. Mol. Microbiol. 59, 923–935. 10.1111/j.1365-2958.2005.04953.x 16420361

[B30] Kalkus J. Dörrie C. Fisher D. Reh M. Schegel H. G. (1993). The giant plasmid pHG207 from *Rhodococcus* sp. encoding hydrogen autotrophy: characterization of the plasmid and its termini. J. Gen. Microbiol. 139, 2055–2060. 10.1099/00221287-139-9-2055 8245832

[B31] Kertesz M. A. Thai M. (2018). Compost bacteria and fungi that influence growth and development of *Agaricus bisporus* and other commercial mushrooms. Appl. Microbiol. Biotechnol. 102, 1639–1650. 10.1007/s00253-018-8777-z 29362825

[B32] Kim J. W. Adachi H. Shin-ya K. Hayakawa Y. Seto H. (1997). Apoptolidin, a new apoptosis inducer in transformed cells from Nocardiopsis sp. J. Antibiot. 50, 628–630. 10.7164/antibiotics.50.628 9711255

[B33] Kinashi H. Mori E. Hatani A. Nimi O. (1994). Isolation and characterization of large linear plasmids from lankacidin-producing *Streptomyces* species. J. Antibiot. 47, 1447–1455. 10.7164/antibiotics.47.1447 7844039

[B34] Kinashi H. Shimaji M. (1987). Detection of giant linear plasmids in antibiotic producing strains of *Streptomyces* by the OFAGE technique. J. Antibiot. 40, 913–916. 10.7164/antibiotics.40.913 3610841

[B35] Kinashi H. Shimaji M. Sakai A. (1987). Giant linear plasmids in *Streptomyces* which code for antibiotic synthesis genes. Nature 328, 454–456. 10.1038/328454a0 3614348

[B36] Kirby R. Hopwood D. A. (1977). Genetic determination of methylenomycin synthesis by the SCP1 plasmid of *Streptomyces coelicolor* A3(2). J. Gen. Microbiol. 98, 239–252. 833570 10.1099/00221287-98-1-239

[B37] Lam K. S. (2006). Discovery of novel metabolites from marine actinomycetes. Curr. Opin. Microbiol. 9, 245–251. 10.1016/j.mib.2006.03.004 16675289

[B38] Lee Y. A. Hendson M. Panopoulus N. J. Schroth M. N. (1994). Molecular cloning, chromosomal mapping, and sequences analysis of copper resistance genes from *Xanthamonas campestris pv. juglandis*: homology with blue copper proteins and multicopper oxidase. J. Bacteriol. 176, 767–778. 10.1128/jb.176.1.173-188.1994 PMC205029 8282694

[B39] Li H. W. Zhi X. Y. Yao J. C. Zhou Y. Tang S. K. Klenk H. P.. (2013). Comparative genomic analysis of the genus *Nocardiopsis* provides new insights into its genetic mechanisms of environmental adaptability. PLoS ONE 8:e61528. 10.1371/journal.pone.0061528 23626695 PMC3634020

[B40] Li Y. Q. Li M. G. Li W. Zhao J. Y. Ding Z. G.. (2007). Griseusin, D., a new pyranonaphthoquinone derivative from a alkaphilic Nocardiopsis sp. J. Antibiot. 60, 757–761. 10.1038/ja.2007.100 18277001

[B41] Lin Y. Kieser H. M. Hopwood D. A. Chen C. W. (1993). The chromosome of DNA of *Streptomyces lividans* 66 is linear. Mol. Microbiol. 10, 923–933. 10.1111/j.1365-2958.1993.tb00964.x 7934869

[B42] Logan E. R. Morgan L. G. (1990). The Potential for Biological Effects of Sediment-Sorbed Contaminants Tested in the National Status and Trends Program. NOAA Technical Memorandum NOS/OMA 52, National Oceanic and Atmospheric Administration, National Ocean Service Seattle, WA, 233.

[B43] Malin G. Ladpidot A. (1996). Induction of synthesis of tetrahydropyrimidine derivatives in *Streptomyces* strains and their effect on *Escherichia coli* in response to osmotic and heat stress. J. Bacteriol. 178, 385–395. 10.1128/jb.178.2.385-395.1996 8550457 PMC177669

[B44] Marineo S. Lecat E. Cusimano M. G. Giardina A. Di Caro V. Puglia A. M. (2005). Identification of SPC2^165^, a new SCP2-derived plasmid of *Streptomyces coelicolor* A3(2). Lett. Appl. Microbiol. 41, 350–354. 10.1111/j.1472-765X.2005.01739.x 16162143

[B45] Martin D. (2002). Contaminant Investigations at Salt Plains National Wildlife Refuge Including an Assessment of Confined Animal Feeding Operations. U.S. Department of the Interior, Fish and Wildlife Service, Region.

[B46] Mayfield C. I. Williams S. T. Ruddick S. M. Hatfield H. L. (1972). Studies on the ecology of actinomycetes in soil, IV. Observations on the form and growth of Streptomyces in soil. Soil Biol. Biochem. 4, 79–91. 10.1016/0038-0717(72)90045-4

[B47] Mukhopadhyay R. Rosen B. P. Phung I. T. Silver S. (2002). Microbial arsenic: from geocycles to genes and enzymes. FEMS Microbio. Rev. 26, 311–325. 10.1111/j.1574-6976.2002.tb00617.x 12165430

[B48] Nett M. Ikeda H. Moore B. S. (2009). Genomic basis for natural biosynthetic diversity in the actinomycetes. Nat. Prod. Rep. 26, 1362–1384. 10.1039/b817069j 19844637 PMC3063060

[B49] Nies D. H. (1992). Resistance to cadmium, cobalt, zinc, and nickel in microbes. Plasmid 27, 17–28. 10.1016/0147-619X(92)90003-S 1741458

[B50] Nies D. H. Silver S. (1989). Plasmid-determined inducible efflux is responsible for resistance to cadmium, zinc, and cobalt in Alcaligenes eutrophus. J. Bacteriol. 171, 896–900. 10.1128/jb.171.2.896-900.1989 2914875 PMC209680

[B51] Nikadio H. (2009). Multidrug resistance in bacteria. Annu. Rev. Biochem. 78, 119–146. 10.1146/annurev.biochem.78.082907.145923 19231985 PMC2839888

[B52] Novakova R. Knirschova R. Farkasovsky M. Feckova L. Rehakova A. Mingyar E. (2013). The gene cluster aur1 form the angucycline antibiotic auricin is located on a large linear plasmid pSA3239 in *Steptomyces aureofaciens* CCM 3239. FEMS Microbiol. Lett. 342, 130–137. 10.1111/1574-6968.12095 23373695

[B53] O'Keefe D. P. Harder P. A. (1991). Occurrence and biological function of cytochrome P450 monooxygenases in the actinomycets. Mol. Microbiol. 5, 2099–2105. 1766383 10.1111/j.1365-2958.1991.tb02139.x

[B54] Pandza S. Biuković G. Paravić A. Dadbin A. Cullum J. Hranueli D. (1998). Recombination between the linear plasmid pPZG101 and the linear chromosome of *Streptomyces rimosus* can lead to exchange of ends. Mol. Microbiol. 28, 1165–1176. 10.1046/j.1365-2958.1998.00877.x 9680206

[B55] Persaud D. Jaagumagi R. Hayton A. (1993). Guidelines for the Protection and Management of Aquatic Sediment Quality in Ontario. Ontario Ministry of Environment and Energy, Toronto, Ontari, 27.

[B56] Picardeau M. Vincent V. (1997). Characterization of large linear plasmids in mycobacteria. J. Bacteriol. 179, 2753–2756. 10.1128/jb.179.8.2753-2756.1997 9098076 PMC179027

[B57] Redenbach M. Scheel J. Schmidt U. (2000). Chromosome topology and genome size of selected actinomycetes species. Antonie Van Leeuwenhoek 78, 227–235. 10.1023/A:1010289326752 11386344

[B58] Reeves A. R. Post D. A. Vandem Boom T. J. (1998). Physical-genetic map of the erythromycin-producing organism *Saccharopolyspora erythraea*. Microbiology 144, 2151–2159. 10.1099/00221287-144-8-2151 9720036

[B59] Sakaguchi K. (1990). Invertrons, a class of structurally and functionally related genetic elements that include linear DNA plasmids, transposable elements and genomes of adeno-type viruses. Microbiol. Rev. 54, 66–74. 2157134 10.1128/mr.54.1.66-74.1990 PMC372759

[B60] Scala J. Williams H. H. (1963). A comparison of selenite and tellurite toxicity in *Escherichia coli*. Arch. Biochem. Biophys. 101, 319–324. 10.1016/S0003-9861(63)80019-3 13976481

[B61] Schneider-Bernlohr H. Formicka-Kozlowkska G. Buhler R. von Wartburg J. P. Zeppezaur. (1988). Active-site-specific zinc-depleted and reconstituted cobalt(II)-human liver alcohol dehydrogenase-preparation, characterization, and complexation with NADH and trans-4-(N,N-dimethylamino)-cinnamaldehyde. Eur. J. Biochem. 173, 275–280. 10.1111/j.1432-1033.1988.tb13995.x 3360008

[B62] Shintani M. Sanchezl Z. K. Kimbaral K. (2015). Genomics of microbial plasmids: classification and identification based on replication and transfer systems and host taxonomy. Front. Microbiol. 6:242. 10.3389/fmicb.2015.00242 25873913 PMC4379921

[B63] Silver S. (1996). Bacterial resistance to toxic metal ions – a review. Gene 179, 9–19. 10.1016/S0378-1119(96)00323-X 8991852

[B64] Summers A. O. Jacoby G. A. (1977). Plasmid-determined resistance to tellurium compounds. J. Bacteriol. 129, 275–281. 401494 10.1128/jb.129.1.276-281.1977 PMC234924

[B65] Sun H. H. White C. B. Dedinas J. Cooper R. Sedlock D. M. (1991). Methylpendolmycin, an indolactam from a Nocardiopsis sp. J. Nat. Prod. 54, 1440–1443. 10.1021/np50077a040 1800642

[B66] Suwa M. Sugino H. Mori E. Sasakoa A. Fujii S. Shinkawa H.. (2000). Identification of two polyketide synthase gene clusters on the linear plasmid pSLA2-L in *Streptomyces rochei*. Gene 246, 123–131. 10.1016/S0378-1119(00)00060-3 10767533

[B67] Williams S. T. Lanning S. Wellington E. M. H. (1984). Ecology of actinomycetes, in The Biology of Actinomycetes, eds Goodfellow M. Mordarski M. Williams S. T. (London: Academic Press), 481–528.

